# Supervised Learning Based Hypothesis Generation from Biomedical Literature

**DOI:** 10.1155/2015/698527

**Published:** 2015-08-25

**Authors:** Shengtian Sang, Zhihao Yang, Zongyao Li, Hongfei Lin

**Affiliations:** College of Computer Science and Engineering, Dalian University of Technology, Dalian 116024, China

## Abstract

Nowadays, the amount of biomedical literatures is growing at an explosive speed, and there is much useful knowledge undiscovered in this literature. Researchers can form biomedical hypotheses through mining these works. In this paper, we propose a supervised learning based approach to generate hypotheses from biomedical literature. This approach splits the traditional processing of hypothesis generation with classic ABC model into AB model and BC model which are constructed with supervised learning method. Compared with the concept cooccurrence and grammar engineering-based approaches like SemRep, machine learning based models usually can achieve better performance in information extraction (IE) from texts. Then through combining the two models, the approach reconstructs the ABC model and generates biomedical hypotheses from literature. The experimental results on the three classic Swanson hypotheses show that our approach outperforms SemRep system.

## 1. Introduction

Literature-based discovery (LBD) was pioneered by Swanson in the 1980s and it focuses on finding new relationships in existing knowledge from unrelated literatures and provides logical explanations [1–3]. Swanson's method is to find a bridge that links two conceptually related topics that ought to have been studied together but never have been. For instance, in his initial work [1], Swanson found there are some types of blood disorders in patients with Raynaud's phenomenon (A), such as high blood viscosity (B), and at the same time he found that fish oil (C) can reduce blood viscosity (B) in other literatures. Since no literature had proved the relationship between fish oil (C) and Raynaud's phenomenon (A) at that time, Swanson proposed that fish oil (C) might treat Raynaud's phenomenon (A), and this hypothesis was verified in medical experiments two years later. In this hypothesis, the topic of blood viscosity (B) served as a bridge between the topics of Raynaud's phenomenon (A) and dietary fish oil (C). Swanson summarized this method as ABC model and he has published several other medical discoveries using this methodology [2, 3].

Since Swanson reported that literature-based discoveries are actually possible, many works have contributed more advanced and automated methods for LBD. Most of the early LBD researches adopted information retrieval (IR) techniques to illustrate the effectiveness of the ABC model for LBD. The idea behind these methods is that the higher cooccurrence frequency the two concepts A and B have, the more related they are. By using the statistical characteristics, the ABC model is automatically achieved. Weeber et al. used concept cooccurrence as a relation measure and applied UMLS semantic types for filtering [4, 5]. For example, the semantic type of one of the cooccurring concepts might be set to disease or syndrome and the other to pharmacologic substance; thus only cooccurrences between a disease and a drug are found. Srinivasan [6] developed a system, called Manjal, which uses Medical Subject Headings (MeSH) terms as concepts and term weights instead of simple term frequencies. The system uses an information retrieval measure based on term cooccurrence for ranking. Yetisgen-Yildiz and Pratt [7] developed a system, called LitLinker, which incorporates knowledge-based methodologies with a statistical method. LitLinker marks the terms with* z*-scores and the terms which are larger than a predefined threshold as the correlated terms to the starting or linking term, and the authors evaluate LitLinker's performance by adopting the information retrieval metrics of precision and recall. However, all the methods mentioned above are mainly based on statistical characters. The main issue of the hypothesis generation approach based on cooccurrence is that the extracted relationships lack logical explanations. On one side, some extracted pairs of entities are completely uncorrelated actually but the high cooccurrence frequency shows a strong association between them. On the other side, although two entities have strong semantic correlations, they might not be extracted from literature because of their low cooccurrence frequency in literature. In addition, although these approaches based on cooccurrence succeed in finding intermediate (B) concepts, they provide no insight into the nature of the relationships among such concepts [8].

Recently Hu et al. [9] presented a system called Bio-SARS (Biomedical Semantic-Based Association Rule System), which utilizes both semantic information and association rules to expand the number of semantic types proposed by Weeber, and this system achieves better performance. Miyanishi et al. advanced the concept of semantic similarity between events based on semantic information [10]. Hristovski et al. combined two natural language processing systems, SemRep and BioMedLee, to provide predications, and analysis using predications can support an explanation of potential discoveries [11]. Cohen et al. proposed the Predication-Based Semantic Indexing (PSI) approach to search predications extracted from the biomedical literature by the SemRep system [12]. Cameron et al. presented a methodology that leverages the semantics of assertions extracted from biomedical literature (called semantic predications) along with structured background knowledge- and graph-based algorithms to semiautomatically capture the informative associations originally discovered manually by Swanson [8]. However, all the above hypothesis generation approaches based on semantic information and association rules utilize the semantic extraction tool, SemRep system. And the performance of SemRep is not perfect: its precision, recall, and* F*-score are 0.73, 0.55, and 0.63, respectively [13]. On one side, low recall (55%) means a substantial number of semantic associations between entities will be missing, and on the other side, low precision (73%) means many false semantic associations will be returned.

The aim of our work is to find an effective method to extract semantic relationships from biomedical literature. In this paper, we propose a supervised learning based approach to generate hypotheses from biomedical literature. This approach divides the traditional hypothesis generation model, ABC model, into two machine learning based models: AB and BC models. The AB model is used to determine whether a physiological phenomenon (linking term B) is caused by a disease (initial term A) in a sentence and the BC model is used to determine whether there exists an entity (e.g., pharmacologic substance, target term C) having physiological effects (linking term B) in a sentence. Compared with the concept cooccurrence and grammar engineering-based approaches like SemRep, machine learning based models usually can achieve better performance in information extraction (IE) from texts [14]. In our experiments, the performances of AB and BC models (both are more than 0.76 measured in* F*-score) are much better than that of SemRep (0.63 in* F*-score [13]). Then through combining the two models, the approach reconstructs the ABC model and generates biomedical hypotheses from literatures. The experimental results on the three classic Swanson hypotheses also show that our approach achieves better performance than the SemRep system.

## 2. Related Resources and Tools

### 2.1. Open and Closed Discovery

Weeber et al. summarized that the hypothesis-generating approaches proposed by Swanson are considered open discovery and the testing approaches are considered closed discovery [4]. 

**Figure 1:**
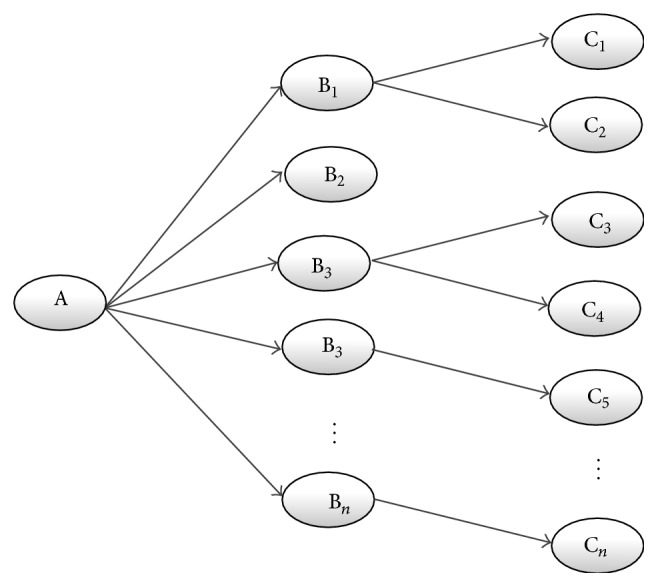
Open discovery process: a one direction search process which starts at A and results in C.

*Open Discovery*. The process of open discovery is characterized by the generation of a hypothesis.  Figure 1 depicts the open discovery approach, beginning with disease A. The researcher will try to find interesting clues (B), typically physiological processes, which play a role in the disease under scrutiny. Next, he (or she) tries to identify C-terms, typically substances, which act on the selected Bs. As a result of the process, the researcher may form the hypothesis that substance C_*n*_ can be used for the treatment of disease A via pathway B_*n*_.

**Figure 2:**
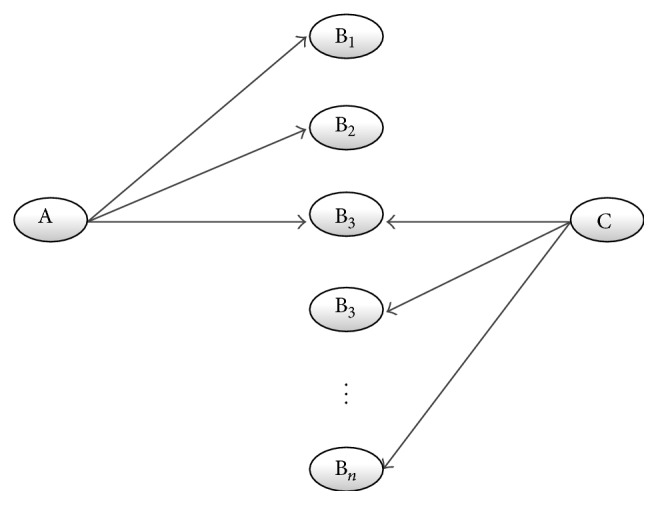
Closed discovery process: the process starts simultaneously from A and C resulting in overlapping Bs.

*Closed Discovery*. A closed discovery process is the testing of a hypothesis. If the researcher has already formed a hypothesis, possibly by the open discovery route described above, he (or she) can elaborate and test it from the literature.  Figure 2 depicts the approach: starting from both disease A and substance C, the researcher tries to find common intermediate B terms.

### 2.2. MetaMap and Semantic Type

MetaMap is a highly configurable application developed by the Lister Hill National Center for Biomedical Communications at the National Library of Medicine (NLM) to map biomedical text to the UMLS Metathesaurus or, equivalently, to identify Metathesaurus concepts referred to in English text. Because every concept in the Metathesaurus has been assigned one or more semantic types, we filter the results of the text-to-concept mapping process by means of the semantic types. In the different stages of the process, we employ different semantic filters. For example, in the stage of selecting intermediate B terms we choose the terms with functional semantic types such as biologic function, cell function, phenomenon or process, and physiologic function. When selecting dietary factors as A terms, we choose the concepts with functional semantic types such as vitamin, lipid, and element, ion, or isotope.  Table 1 provides the functional semantic types that we use to filter the linking terms and target terms in our experiments.

### 2.3. SemRep and SemMedDB

SemRep was developed at the National Library of Medicine and is a program that extracts semantic predications (subject-relation-object triples) from biomedical free text. For example, from the sentence in Table 2, SemRep extracts four predications (as shown in Table 2). Semantic Medline Database (SemMedDB) is a repository of semantic predications extracted by SemRep. SemMedDB currently contains information about approximately 70 million predications from all of PubMed citations (more than 23.5 million citations, as of April 1, 2014) and forms the backbone of Semantic Medline application [15].

### 2.4. General Concepts Filtering

Many general concepts will be generated after the processing of Named Entity Recognition (NER) by MetaMap. General concepts refer to the terms which have relationships with many entities but have meaningless concept, such as “disease.” Because the existence of general concepts may reduce the effect of knowledge discovery, they need to be filtered. In our experiment, first we extract all the sentences with relations such as “subject ISA object” from SemMedDB and then a general concept list is constructed by collecting all the objects from the sentences. All the relations used in our experiment are PART_OF, LOCATION_OF, and ISA.

### 2.5. Stanford Parser

The Stanford Parser was built by the Stanford NLP (natural language processing) Group in the 1990s. It is a program that works out the grammatical structure of sentences, for instance, which groups of words go together (as “phrases”) and which words are the subject or object of a verb. The primary function of the Stanford Parser is to analyze and extract the syntactic structure of sentences and part-of-speech tagging (POS tagging). The parser provides Stanford Dependencies output as well as phrase structure trees. In our method, the features of the graph kernel-based method are extracted by the Stanford Parser [17].

## 3. Method

For knowledge discovery, we split the traditional ABC model into two models—AB model and BC model. Both models are constructed by using cotraining methods. The purpose of AB model is to determine whether a physiological phenomenon (linking term) is caused by a disease (initial term) in a sentence, and the BC model is used to judge whether there exists an entity (target term) having physiological effects (linking term) on human beings in a sentence.

The supervised learning methods used in our experiment are all kernel-based methods, and kernel methods are effective alternatives to explicit feature extraction [18]. They retain the original representation of objects and use the object only via computing a kernel function between a pair of objects. Such a kernel function makes it possible to compute the similarity between objects without enumerating all the features. And the features we employ in our experiment are as follows.

### 3.1. Feature Sets

A kernel can be thought of as a similarity function for pairs of objects. The following features are used in our feature-based kernel.

*(i) Neighboring Words*. In a sentence, the words surrounding two concepts have a significant impact on the existence of the relationship between the two concepts. In our method, the words surrounding two concepts are considered the neighboring words feature. This feature consists of all the words that are located between the two concepts, words surrounding two protein names, which include three words to the left of the first protein name and three words to the right of the second protein name. We add a prefix to each word to distinguish the different positions of the words. For example, the word “word” is expressed in the above three cases as “m_word,” “l_word,” and “r_word,” respectively. When using the neighboring word feature, a dictionary of the whole corpus will be established, and a sentence is represented as a Boolean vector, where “1” means the feature exists in the sentences and “0” means the feature does not exist in the sentence.

*(ii) Entity Name Distance*. Under the assumption that the shorter the distance (the number of words) between two entity names is, the more likely the two proteins have interaction relation, the distance is chosen as a feature. The feature value is set as the number of words between two entity names.

*(iii) Relationship Words*. Through analyzing the corpus, we make the hypothesis: if there exists a relationship between two entity concepts, there will be a greater probability that some verbs or their variants appear surrounding the two concepts, such as “activation,” “induce,” and “modulate.” These words are used as relationship words in our method. We build a relationship words list of about 500, and the list is used to determine whether there is a relationship between the two entity concepts in the sentences. Boolean “1” means the relationship word exists in the sentence and “0” means it does not exist.

*(iv) Negative Words*. Some negative words such as “not,” “neither,” and “no” exist in some sentences, and these negative words express that there is no relationship between two entity concepts. If a negative word and a relationship word cooccur in a sentence, it is difficult to judge whether there exists a relationship in the sentences only based on relationship words, so negative words features were introduced to improve the situation. Boolean “1” means the negative word exists in the sentence and “0” means it does not exist.

For example, in sentence A, “Quercetin, one of the most representative C0596577 compounds, is involved in antiradical, C0003402, and prooxidant biological processes,” all the features extracted from it are shown in Table 3 (we preprocessed sentence A with MetaMap, and the two target entities are represented by their CUIs). Finally, the sentence will be represented by a feature vector.

### 3.2. Graph Kernel

**Figure 3:**
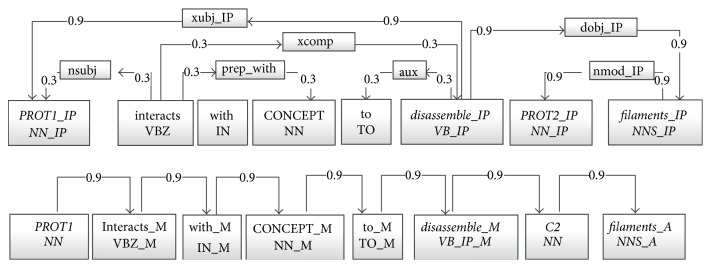
Graph representation generated from an example sentence. The candidate interaction pair is marked as PROT1 and PROT2; the third protein is marked as PROT. The shortest path between the proteins is shown in bold. In the dependency based subgraph all nodes in a shortest path are specialized using a posttag (IP). In the linear order subgraph possible tags are before (B), middle (M), and after (A). For the other two candidate pairs in the sentence, graphs with the same structure but different weights and labels would be generated.

In our experiment, all the sentences are parsed by Stanford Parser to generate the output of dependency path and POS path. A graph kernel calculates the similarity between two input graphs by comparing the relations between common vertices (nodes). The graph kernel used in our method is the all-paths graph kernel proposed by Airola et al. [19]. The kernel represents the target pair using graph matrices based on two subgraphs, where the graph features include all nonzero elements in the graph matrices. The two subgraphs are a parse structure subgraph (PSS) and a linear order subgraph (LOS), as shown in Figure 3. PSS represents the parse structure of a sentence and includes word or link vertices. A word vertex contains its lemma and its POS, while a link vertex contains its link. Additionally, both types of vertices contain their positions relative to the shortest path. LOS represents the word sequence in the sentence and thus has word vertices, each of which contains its lemma, its relative position to the target pair, and its POS.

For the calculation, two types of matrices are used: a label matrix *L* and an edge matrix *A*. The label matrix is a (sparse) *N* × *L* matrix, where *N* is the number of vertices (nodes) and *L* is the number of labels. It represents the correspondence between labels and vertices, where *L*_*ij*_ is equal to 1 if the *i*th vertex corresponds to the *j*th label and 0 otherwise. The edge matrix is a (sparse) *N* × *N* matrix and represents the relation between pairs of vertices, where *A*_*ij*_ is a weight *w*_*ij*_ if the *i*th vertex is connected to the *j*th vertex and 0 otherwise. The weight is a predefined constant whereby the edges on the shortest paths are assigned a weight of 0.9 and other edges receive a weight of 0.3. Using the Neumann Series, a graph matrix *G* is calculated as

(1) $$\begin{array}{c} G=L^{T}\overset{\infty }{\underset{n=1}{\sum }}A_{n}L=L^{T}\left(\;{\left(\;I-A\;\right)}^{-1}-I\;\right)L. \end{array}$$

This matrix represents the sums of all the path weights between any pair of vertices resulting in entries representing the strength of the relation between each pair of vertices. Using two input graph matrices *G* and *G*′, the graph kernel *k*(*G*, *G*′) is the sum of the products of the common relations' weights, given by

(2) $$\begin{array}{c} k\left(\;G,G^{\prime }\;\right)=\overset{L}{\underset{i=1\;}{\sum }}\overset{L}{\underset{j=1}{\sum }}G_{ij}G_{ij}^{\prime }. \end{array}$$

### 3.3. Cotraining Algorithm

Cotraining is a semisupervised learning algorithm used when there are only small amounts of labeled data and large amounts of unlabeled data, and it requires two views of the data [20]. It assumes that each example is described using two different feature sets that provide different, complementary information about the instance. Cotraining first learns a separate classifier for each view using any labeled examples. The most confident predictions of each classifier on the unlabeled data are then used to iteratively construct additional labeled training data. In our experiment, the two different feature sets used to describe a sentence are word features and grammatical features (graph kernel extracted by Stanford Parser), respectively.

## 4. Classification Models

### 4.1. Evaluation Metrics

In our study, the evaluation metrics precision (*P*), recall (*R*), and *F*-score (*F*) are employed, which are defined as follows:

(3) P=TPTP+FP∗100%,R=TPTP+FN∗100%,F=2∗P∗RP+R∗100%.

TP is the number of correctly predicted pieces of data, FP is the number of false positives, and FN is the number of false negatives in the test set. *P* is used to evaluate the accuracy of a model, *R* is recall rate, and *F* is the harmonic mean of precision and recall rates.

In addition, we define the effective linking terms as the terms in a sentence which can connect the initial term and the target term. For example, in the case of* Raynaud's disease and fish oil*, the concepts of* platelet aggregation*,* blood vessel contraction* are all effective linking terms. The definition of the proportion of the effective linking terms in all linking terms is as follows:

(4) $$\begin{array}{c} S=\frac{n}{N}*100\%, \end{array}$$

where *n* is the number of effective linking terms and *N* is the number of all the terms which have relationships with initial terms.

### 4.2. Training AB Model

The purpose of the AB model is to determine whether a physiological phenomenon (linking term) is caused by a disease (initial term) in a sentence.

We obtain all the sentences (the corpus) used in our experiment through searching from Semantic Medline Database with 200 different semantic types about “disease or syndrome” defined by MeSH (Medical Subject Headings). Then all the sentences are processed with the MetaMap for Named Entity Recognition (NER) and to limit the semantic types of initial terms and linking terms in a sentence, we filter out the sentences which contain the concepts of the unneeded semantic types. Finally we obtain a total of 20,895 sentences and we randomly choose only 1,000 sentences (probably 5% of all the sentences) for manual annotation; then we build two initial labeled data sets as initial training set T_initial_ (500 labeled sentences) and test set (other 500 labeled sentences), respectively. There are two reasons why we randomly choose 1,000 sentences for manual annotation: first, manual annotation is very time consuming and expensive, and at the same time unlabeled training data are usually much easier to obtain. This is also why we introduce the cotraining method to improve the performance of our experiment. And the other reason is when cotraining style algorithms are executed, the number of labeled examples is usually small [21], so we select about 5% of the corpus as training set and test set, and the other 95% of the corpus is used for extending the training set later.

During the manual annotation, the following criteria are met: if a sentence contains a relationship belonging to the semantic type list (see Table 1) between two entity concepts, we think there is a positive correlation between the two entities and label the sentence as a positive example. In addition, some special relationships such as “B in A” and “A can change B” are also classified as positive examples since they mean a physiological phenomenon (B) occurs when someone has the disease (A). If there is no obvious relationship in a sentence and only a cooccurrence relationship, we label it as a negative example. For the patterns such as “A is a B” and “A and B”, we label them as negative examples since “A is a B” is “IS_A” relationship and “A and B” is a coordination relationship, and they are not the relationship we need. When the process was completed, we estimated the level of agreement. Cohen's kappa [22] score between each annotator, as shown in Table 4, is 0.8664, and content analysis researchers generally think of a Cohen's kappa score more than 0.8 as good reliability [22]. Comparably, Light et al. achieved their highest kappa value (0.68) in their manual annotation experiments on Medline abstracts [16].

**Figure 4:**
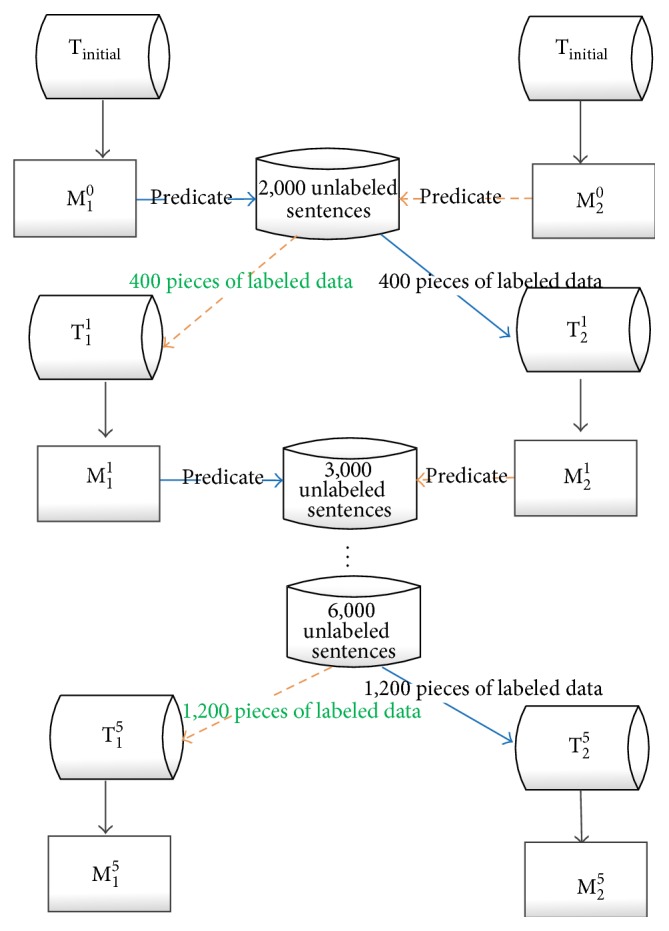
The process of training AB model.

As shown in Figure 4, the process of training AB model is as follows: at first, two initial SVM classifiers, M_1_^0^ (graph-based kernel) and M_2_^0^ (feature-based kernel), are trained using initial training set T_initial_ which contains 500 labeled examples, and the test results of two classifiers are as follows: with classifier M_1_^0^, the values of precision, recall, and* F*-score are 72.88%, 83.33%, and 77.76%, respectively. And at the same time, the results of classifier M_2_^0^ are 74.35%, 76.33%, and 75.33%, respectively.

In the next step, 2,000 unlabeled sentences (a section of the unlabeled corpus) are predicated by classifiers M_1_^0^ and M_2_^0^, respectively. The classifier M_1_^0^ selects the top 200 scores (10% of 2,000 results classified by M_1_^0^) as the positive examples and the bottom 200 scores as the negative examples; then we put these 400 newly labeled examples into the data set T_initial_ (500) as a new training set T_2_^1^ (900 examples). And at the same time, the classifier M_2_^0^ selects the top 200 scores (10% of 2,000 results classified by M_2_^0^) as the positive examples and the bottom 200 scores as the negative examples; then we put these 400 newly labeled examples into the data set T_initial_ (500) as a new training set T_1_^1^ (900 examples). Then two new classifiers M_1_^1^ and M_2_^1^ are trained from T_1_^1^ and T_2_^1^, respectively. After that, 3,000 unlabeled sentences (a section of the unlabeled corpus) are classified by M_1_^1^ and M_2_^1^, respectively. M_1_^1^ selects the top 300 scores (10% of 3,000 results classified by M_1_^1^) as positive examples and the bottom 300 scores as negative examples. We use the 600 newly labeled examples to update the T_2_^1^ and then we obtain a new training set T_2_^2^, while M_2_^1^ also selects 600 newly labeled examples to update T_1_^1^ and then we obtain the new training set T_1_^2^. Such a process is repeated for a preset number of learning rounds.

In our experiment, the preset number of learning rounds is five and the sizes of corresponding extended set are 2,000, 3,000, 4,000, 5,000, and 6,000, respectively. And the size of training set increases from the initial 500 (T_initial_) to 900 (T_1_^1^ and T_2_^1^), 1,500 (T_1_^2^ and T_2_^2^), 2,300 (T_1_^3^ and T_2_^3^), 3,300 (T_1_^4^ and T_2_^4^), and 4,500 (T_1_^5^ and T_2_^5^), respectively. There are two reasons why we set five times of learning rounds. First, after five learning rounds, there are no extra unlabeled sentences. At first 95% of the corpus (20,895) is used for extending the training set, and after five times of expansion of the training set, there are no extra unlabeled data. And the second reason is the cotraining process could not improve the performance further after a number of rounds [21], and we should terminate cotraining on an appropriate round to avoid some wasteful learning rounds.

**Figure 5:**
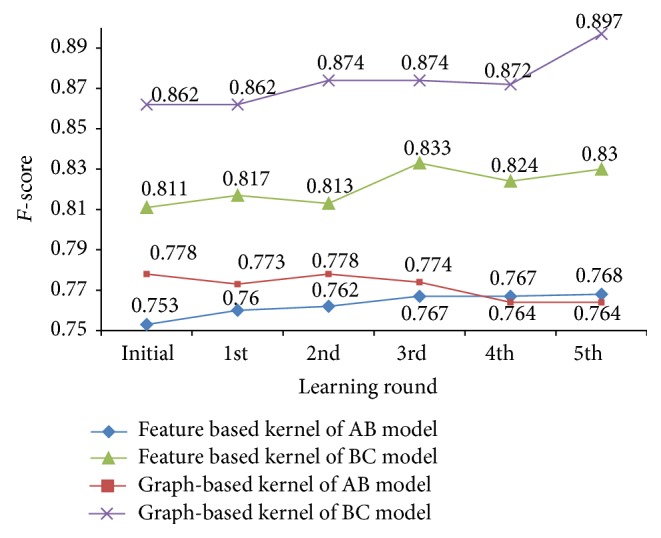
The results of training AB and BC models of every learning round by cotraining algorithms.

The best testing result is from the second learning round (the classifier is M_1_^2^ (graph kernel) and the training set is T_1_^2^): an* F*-score of 77.8% is achieved as shown in Figure 5. After five rounds of cotraining learning, although there are fluctuations, the* F*-score of feature-based kernel achieves a continuous improvement and finally reaches 76.82%.

We can know from both figures that the best results of two models (feature-based model and graph kernel-based model) are all better than the results of the model (M_1_^0^ and M_2_^0^) only trained by the initial training set (T_initial_). The results show that it is feasible to adopt a cotraining algorithm to improve the effectiveness of extracting relationships from a sentence. Meanwhile, since the model based on graph kernel outperforms the feature-based model, we choose the former as our AB model in the following experiments. In our experiments, we choose M_1_^2^ as the AB model.

### 4.3. Training BC Model

The BC model is used to determine whether there exists an entity (target term) having physiological effects (linking term) on human beings in a sentence. The process of training the BC model is similar to the process of training the AB model.

At first, as with the steps in the process of training the AB model, we obtain 20,490 unlabeled sentences from SemMedDB as corpus, and we randomly choose 1,000 sentences (probably 5% of the corpus) to construct the initial training set T_initial_ (500) and test set (500). The process of training BC model is the same to the process of training the AB model. The preset number of learning rounds is five and the sizes of corresponding extended set are 2,000, 3,000, 4,000, 5,000, and 6,000, respectively.

The best result is achieved in the fifth cotraining learning round (the classifier is M_2_^5^ (graph kernel) and the training set is T_2_^5^), and the value of* F*-score is 89.71% as shown in Figure 5. And the highest* F*-score (83.3%) of feature-based kernel is achieved in the third cotraining learning round.

Like the results of the AB model, the best results of two models (feature-based model and graph kernel-based model) are all better than the results of the model (M_1_^0^ and M_2_^0^) only trained by the initial training set (T_initial_). It proves the validity of cotraining method again. Meanwhile, since the model based on graph kernel outperforms the feature-based model, we choose the former as our BC model in the following experiments. In our experiments, we choose M_1_^5^ as the BC model.

In both experiments, the best results of graph kernel outperform feature-based model; the reason is that not only do the features selected by graph kernel include most parts of the features adopted by feature kernel, but also the graph kernel approach captures the information in unrestricted dependency graphs which combines syntactic analysis with a representation of the linear order of the sentence and considers all possible paths connecting any two vertices in the resulting graph.

## 5. Hypothesis Generation and Discussion

After the AB and BC models have been built, we combine them to reconstruct the ABC model and validate the three classic Swanson's hypotheses, that is, “Raynaud's disease and fish oil,” “migraine and magnesium,” and “Alzheimer's disease and indomethacin.” We compare the results generated by our method with the results generated by using SemRep Database in closed discovery and open discovery processes, respectively. In our study, the result of hypothesis discovery achieved by SemRep is presented as a benchmark.

### 5.1. Closed Discovery Experiment

In our study, the hypothesis* Raynaud's disease and fish oil* is used as an example to verify the effectiveness of our method. First, the initial terms are set to “Raynaud's disease” and “Raynaud's phenomenon” and the target terms are set to “fish oil” and “eicosapentaenoic acid” (an important active ingredient of fish oil). The initial terms are used as keywords to retrieve all the sentences from the Medline Database, respectively (the time is limited before 1986 since Swanson found the classic hypothesis in 1986, and from then on the hypothesis is considered public knowledge). Then we obtain all the sentences that contain either the initial terms or target terms, and then all the sentences are processed by MetaMap.

Secondly, we filter out the sentences which do not contain any concept belonging to semantic type list or contain the concepts in the general concept list. Then the AB model is adopted to classify all the sentences containing the initial terms and at the same time we use BC model to classify all the sentences containing the target terms, and finally we obtain the effective linking terms by taking the intersection of two sets of positive examples (one set is from the AB model and the other is produced by the BC model).

**Figure 6:**
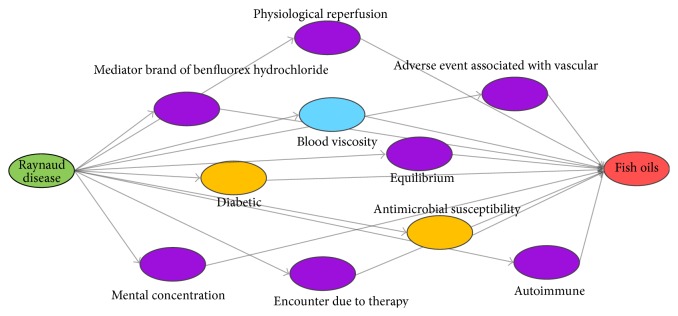
The result of closed discovery of “Raynaud's disease and fish oils.” The purple nodes in the figure are the linking terms discovered only by our method; the yellow nodes represent the linking terms found only by SemRep Database; and the blue node represents the terms found by both methods.

The only difference between the SemRep method and our method is that its effective linking terms are obtained by taking the intersection of two sets of linking terms (the two sets of linking terms are all retrieved from SemRep instead of using the AB model and BC model, and one set is retrieved with the initial terms and the other is retrieved with the target terms).  Figure 6 shows the result obtained by the two methods. In addition, the other two classic Swanson's hypotheses* migraine and magnesium* and* Alzheimer's disease and indomethacin* are also verified with our method and the results are shown in Table 5, and part of the effective linking terms discovered by our method are shown in Table 6.

As can be seen from Table 5, compared with the results obtained by SemRep method, our method significantly improves the number and proportion of effective linking terms. The ratios of our method are much higher than those of SemRep method, and more effective linking terms mean more possible useful hypothesis is generated. For example, Figure 6 shows the potential principles in treatment of “Raynaud's disease with fish oils” discovered by two methods. The purple nodes in the figure are the linking terms discovered only by our method; the yellow nodes represent the linking terms found only by SemRep Database; and the blue node represents the terms found by both methods.  Figure 6 shows that our method not only found the linking term “blood viscosity” also found by SemRep, but also found more effective linking terms. For example, the intermediate purple node “adverse event associated with vascular” was found by our approach from the abstracts [PMID 2340647] and [PMID 6982164]. From [PMID 2340647] our approach extracted “dietary fish oil inhibits vascular reactivity,” and at the same time from [PMID 6982164] we extracted “vasodilation inhibits Raynaud syndrome”, then we may make the hypothesis that dietary fish oil treats Raynaud's syndrome by inhibiting vascular reactivity (i.e., vasoconstriction, vascular constriction (function)) which causes Raynaud's disease, and this hypothesis has been verified in medical experiments [23].

The reason why our method can discover more effective linking terms than SemRep is that, compared with grammar engineering-based approaches like SemRep, machine learning based models usually can achieve better performance in information extraction from texts. In our experiments, the performances of our machine learning based AB and BC models are much better than that of SemRep which means our method can return more effective linking terms and, therefore, it may help find more potential principles in hypothesis generation.

### 5.2. Open Discovery Experiment

In this paper, the process of finding the hypothesis* migraine and magnesium* is used as an illustration to explain the procedure of open discovery.

**Figure 7:**
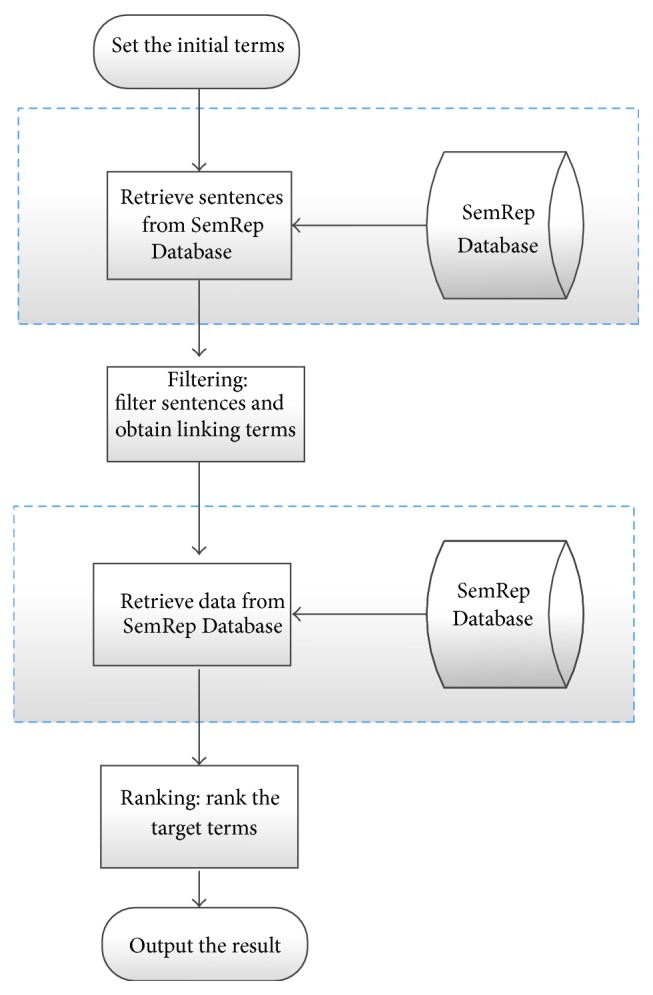
The procedure of open discovery with SemRep Database.

**Figure 8:**
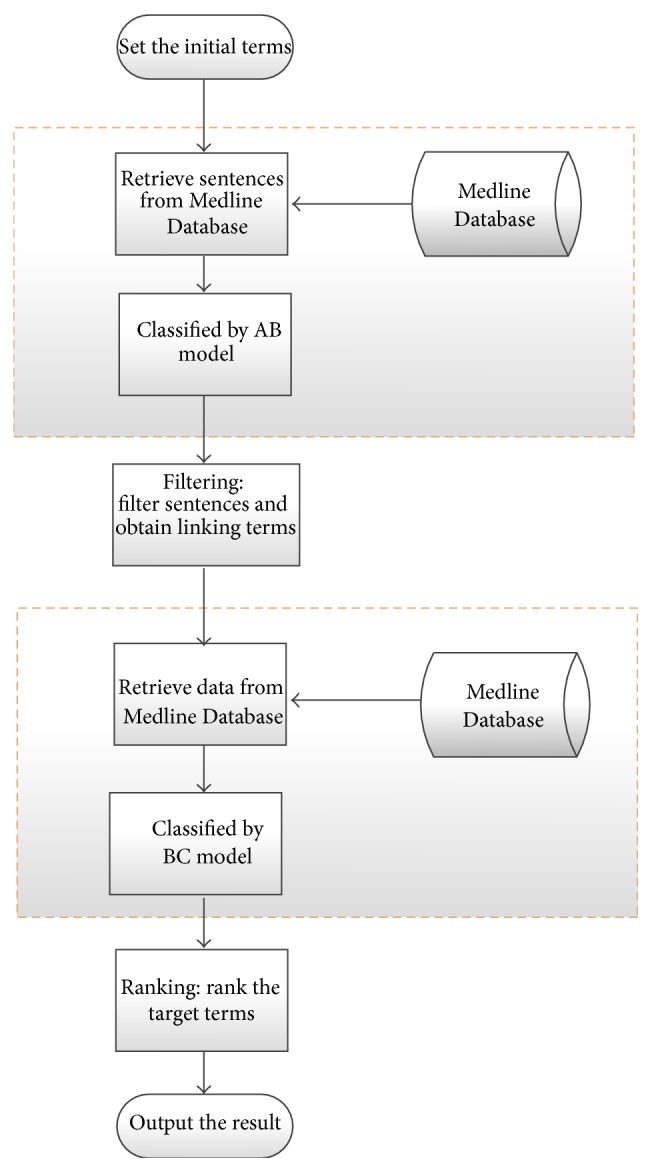
The procedure of open discovery with our method.

The processes of open discovery with different methods are shown in Figures 7 and 8, respectively. In the process of open discovery by using SemRep Database (Figure 7), first the initial terms (A terms) are specified by providing concept “Migraine Disorder,” which is used as a keyword to retrieve data from the SemRep Database. Then we obtain all the sentences which contain both initial terms (Migraine Disorder) and linking terms (other entities). Second, we filter all the sentences obtained from step one, and the specific rules are the same as we mentioned in closed discovery (we filter out the sentences which do not contain any concept belonging to semantic type list or contain the concepts in broad concept list). The third step is similar to the first step, all the linking terms (extracted from every sentence from the filter step) are used as keywords to retrieve data from the SemRep Database; then we get all the sentences which contain both linking terms (B terms) and target terms (C terms). At last we rank all the target terms and output the results. The scoring rules are applied to rank the target terms [24].

The process of open discovery with the AB-BC model is shown in Figure 8. The differences between the two processes are shown in the blue dashed boxes (Figure 7) and yellow dashed boxes (Figure 8). Instead of obtaining all the sentences from the SemRep Database (as shown in blue dashed boxed in Figure 8), two steps are applied in our method to obtain the data: first we obtain raw sentences from the Medline Database, and in the second step all the sentences are classified by either AB model or BC model; then we collect the set of positive example as experimental data. Another processing (e.g., filtering and ranking) is the same with that of the SemRep method.

The processing of open discovery with AB-BC model is shown in Figure 8. The differences between the two processes are shown in the blue dashed boxes (Figure 7) and yellow dashed boxes (Figure 8). Instead of obtaining all the sentences from the SemRep Database (as shown in blue dashed boxed in Figure 7), two steps are applied in our method to obtain the data: first we obtain raw sentences from the Medline Database, and in the second step all the sentences are classified by either AB model or BC model; then we collect the set of positive examples as experimental data. And other procedures (filtering and ranking) are the same with the SemRep method.

The results of open discovery are shown in Table 7. The value of the number to the right of the slash is the total number of potential target terms we obtained, and the number to the left of the slash is the ranking of the target term we really want to obtain. For example, from the rediscovery of* migraine-magnesium* association, we obtain 535 potential target terms with SemRep method and the ranking of* magnesium* is 186 while 5,349 target terms are discovered with our method and the ranking of* magnesium* is 97. As can be seen from the above results, our method not only can obtain more potential target terms but also can get higher ranking of the real target terms.

The higher the ranking of the real target terms is, the more valuable the results become. And more potential target terms mean more clues about the pathophysiology of disease, for example, from the rediscovery of* Alzheimer's disease and indomethacin* hypothesis, 650 and 2,639 potential target terms are found by SemRep method and our method, respectively. Although neither of two methods finds the real target term* indomethacin* (*indomethacin* is a nonsteroidal anti-inflammatory drug (NSAID) commonly used as a prescription medication to reduce fever, pain, stiffness, and swelling), both methods find another NSAID* indoprofen*, and many epidemiological studies have supported the hypothesis that chronic intake of NSAID is associated with a reduced risk of* Alzheimer's disease* [25–27]. Moreover, many other NSAIDs have been found by our method such as* carprofen*,* Proxen,* and* aspirin*, and the result of our method finds more clues about the pathophysiology of Alzheimer's disease:* prostaglandins* [28–30] and* thromboxane* [31]. The detailed results are shown in Table 8.

The results of the experiment with our method outperform almost all the results of SemRep method. In addition, we found that combining the results of two methods can further improve the performance. For example, in the rediscovery of physiopathological hypothesis of* Raynaud's phenomenon*, we obtain 380 potential target terms with SemRep method and 5,762 potential target terms with our method. Although our method finds many more potential target terms than SemRep method, sometimes the result of SemRep method has a higher ranking (68) than ours (230).

Therefore, we took the following steps to combine the results of two methods: first, we obtain the result sets returned by SemRep and our methods, respectively. Then we retain the intersection of two result sets. For a term in the intersections, its score is set as the sum of its scores in the original result sets and ranked with its score. The detailed results are also shown in Table 7, and it can be seen that the results from “combined result set” have higher ranking than both original methods. The reason is that in the intersection set of two result sets many potential target terms are eliminated, and, at the same time, the real target terms will not be eliminated since such terms usually have real association with the initial term and, therefore, will be retained by both methods.

## 6. Conclusions

Biomedical literature is growing at an explosive speed, and researchers can form biomedical hypotheses through mining this literature.

In this paper, we present a supervised learning based approach to generate hypotheses from biomedical literature. The approach splits the classic ABC model into AB and BC models and constructs the two models with a supervised learning method, respectively. The purpose of the AB model is to determine whether a physiological phenomenon (linking term) is caused by a disease (initial term) in a sentence, and the BC model is used to judge whether there exists an entity (target term) having physiological effects (linking term) on human beings in a sentence. Compared with the concept cooccurrence and grammar engineering-based approaches like SemRep, machine learning based AB and BC models can achieve better performance in mining association between bioentities from texts.

In addition, the cotraining algorithm is introduced to improve the performance of the two models. Then through combining the two models, the approach reconstructs the ABC model and generates biomedical hypotheses from literature.

The experimental results on the three classic Swanson's hypotheses show that our approach can achieve better performance than SemRep. This means our approach can discover more potential correlations between the initial terms and target terms, and, therefore, it may help find more potential principles for the treatment of certain diseases.

## Figures and Tables

**Table 1:** The semantic type filter used in our experiments.

Linking term	Target term
Biologic function	Organic chemical
Cell function	Lipid
Finding	Pharmacologic substance
Molecular function	Vitamin
Organism function	Element, ion, or isotope
Organ or tissue function	
Pathologic function	
Phenomenon or process	
Physiologic function	

**Table 2:** The semantic type filter used in our experiments.

Sentence	We used hemofiltration to treat a patient with digoxin overdose that was complicated by refractory hyperkalemia

Extracted predications (subject-relation-object triples)	Hemofiltration-TREATS-Patients
	Digoxin overdose-PROCESS_OF-Patients
	Hyperkalemia-COMPLICATES-Digoxin overdose
	Hemofiltration-TREATS(INFER)-Digoxin overdose

**Table 3:** Features extracted from example sentence A.

Feature names	Feature values
Left words	l_the, l_most, l_representative
Words between two entity names	m_compounds, m_is, m_involved, m_in, m_antiradical
Right words	r_and, r_prooxidant, r_biological
Relationship words	involved
Protein name distance	5
Negative word	—

**Table 4:** Annotation values of two annotators.

Reviewer 2	Reviewer 1		
	Positive	Negative	Kappa score
Positive	385	22	
Negative	43	550	
Ours			0.8664
Light et al. [16]			0.68

**Table 5:** The number and scale of the useful linking concept.

Hypothesis	Methods	Effective linking terms/linking terms	Ratio
Raynaud's disease and fish oils	Our method	10/106	9.43%
	SemRep	3/132	2.2%

Migraine and magnesium	Our method	67/297	22.56%
	SemRep	10/187	5.35%

Alzheimer's disease and indomethacin	Our method	47/251	18.72%
	SemRep	9/87	10.34%

**Table 6:** Part of the effective linking terms discovered with our method.

Raynaud's disease and fish oils	Migraine and magnesium	Alzheimer's disease and indomethacin
Blood viscosity Adverse event associated with vascular Mental concentration Component of protein Autoimmune Mediator brand of benfluorex hydrochloride Physiological reperfusion	Arteriospasm Vasodilation Desiccated thyroid Homovanillate Beta-adrenoceptor-activity Regional blood flow Recognition (psychology) Container status identified Hydroxide ion Muscular dystrophy	Metabolic rates Pituitary hormone-preparation Desiccated thyroid Cerebrovascular-circulation Normal blood pressure P blood group antibodies Dopamine hydrochloride HLA-DR antigens Pentose phosphate-pathway Toxic epidermal-necrolysis

**Table 7:** Result of open discovery.

Initial terms	SemRep	Our method	Combined result set
Raynaud disease and eicosapentaenoic acid	68/380	230/5762	61/246
Migraine disorders and magnesium	186/535	97/5349	25/297
Alzheimer's disease and indomethacin	—/650	—/2639	—/275
Alzheimer's disease and indoprofen	373/650	250/2639	38/275

**Table 8:** More target terms about Alzheimer's disease.

Target terms	Alzheimer's disease
Asprin	1370/2639
Thromboxane	1455/2639
Carprofen	2291/2639
Prostaglandins	2488/2639
